# Angiotensin II treatment in COVID-19 patients: more risk than benefit? A single-center experience

**DOI:** 10.1186/s13054-020-03143-7

**Published:** 2020-07-09

**Authors:** Ulrike Heinicke, Elisabeth Adam, Michael Sonntagbauer, Andreas von Knethen, Kai Zacharowski, Holger Neb

**Affiliations:** 1 Department of Anesthesiology, Intensive Care Medicine and Pain Therapy, University Hospital Frankfurt, Frankfurt am Main, Germany; 2 Fraunhofer – IME, Project Group Translational Medicine and Pharmacology (TMP), Frankfurt am Main, Germany

Critical care treatment of severely affected COVID-19 patients remains extremely challenging. To date, no experimental intervention has shown a significant benefit in this state of disease. Intravenous application of angiotensin II (ATII) has been postulated in cases with vasodilatory shock, as ATII is already known to be a very potent vasoconstrictor and has been used in patients suffering from septic shock [1]. To assess a potential deficiency of serum ATII, we used dipeptidyl-peptidase 3 (DPP3) levels, measurement provided by sphingotec (Nexus IB 10, sphingotec GmbH, Hennigsdorf, Germany), to guide ATII administration in severe COVID-19 patients. DPP3 is a ubiquitous peptidase that cleaves ATII enzymatically to angiotensin 4 and further to C-terminal tetrapeptide sequence. High serum level concentrations of DPP3 might lead to a lack of serum ATII and are also suggested to be a marker for cell death [2]. DPP3 levels elevated above 40 ng/ml are supposed to be associated with multi-organ failure and short-term death. We therefore initiated compassionate use treatment with ATII; initially four of in total six severe COVID-19 patients in vasodilatatory shock and DPP3 levels exceeding 40 ng/ml were treated with this strategy. Treatment of vasodilatatory shock prior to ATII infusion had been initiated with a combination of noradrenaline and vasopressin infusion. In all cases, the dosage of noradrenaline and vasopressin infusion was reduced after initiation of ATII infusion. However, the overall response rate was poor, as 3 of the first 4 patients died during or shortly after ATII administration. While one patient died due to severe intracerebral hemorrhage, one other died of a septic shock caused by mesenterial ischemia. The third patient died being in total multi-organ failure. After initial ATII use in distraught situations in these four patients, we hoped that initiation of ATII infusion in severe COVID-19 patients without massive vasodilatory shock might be favorable. However, after the death of another two patients (patients 5 and 6) with severe COVID-19 due to total respiratory failure, we abandoned ATII treatment in COVID-19 patients all together. Ethics committee approved the post hoc analysis of these data for scientific purposes.

Patient characteristics and inflammation markers such as interleukin-6, amongst others, are shown in Figs. 1 and 2. ATII infusion differed in dose per time per patient, aiming to maintain a mean arterial blood pressure > 65 mmHg as main target. Murray score was used to quantify the level of lung failure. In our cases, Murray score was not affected by ATII infusion, foiling expectations generated by the recent publication of Zangrillo et al. [3]. AT II did not improve any of our obtained parameters in an obvious manner. As COVID-19 progresses, patients seem to be in a different state of illness, in which progressive tissue damage is not a direct consequence of the infection itself. More likely, a dysregulated cardiovascular system could be the reason for the course we observe in severe COVID-19 cases. This dysregulation might be the consequence of a mismatch of ATII and angiotensin converting enzyme receptor 2, possibly caused by a dysregulated immune system. In this state, the effects of additional ATII could be devastating, as ATII is known to aggravate inflammation, fibrotic reconstruction, and vascular permeability [4]. These considerations and our experience with the administration of intravenous ATII in six COVID-19 patients do not support the use of this vasopressor. The use of a potent vasoconstricting agent like ATII at earlier stages of COVID-19 without vasodilatatory shock is limited due to the risk of hypertensive episodes. We did not observe the previously postulated antiviral effects of ATII [5]. ATII treatment was not successful in our hands when treating COVID-19 patients.

**Fig. 1:**
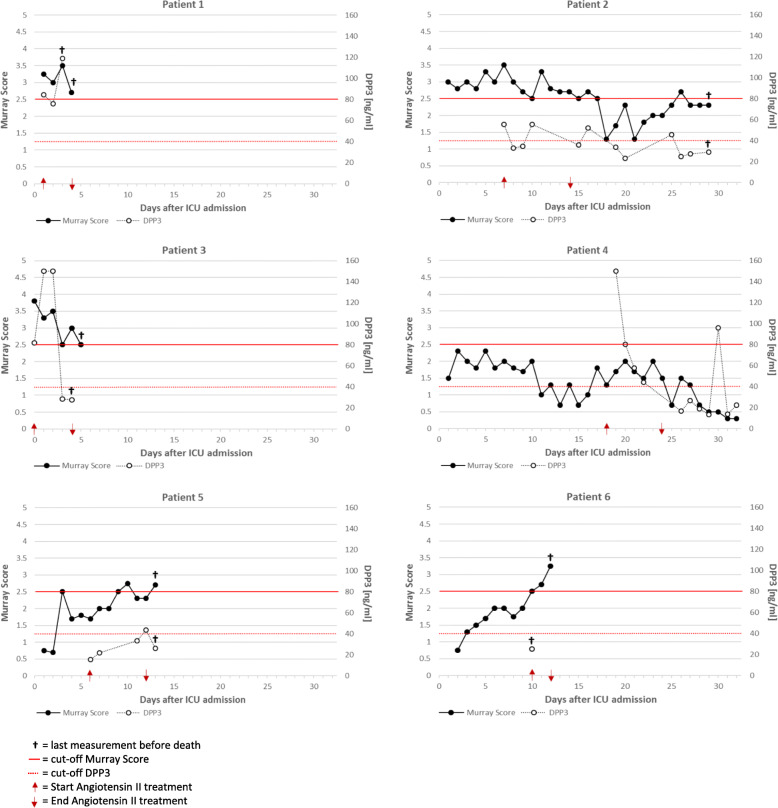
Murray score and DPP3 levels in angiotensin II-treated ICU patients. Assessment of Murray score and dipeptidyl-peptidase 3 (DPP3) levels in COVID-19-positive ICU patients during angiotensin II treatment. The Murray score was calculated by scoring hypoxemia, respiratory system compliance, chest radiographic findings, and level of positive end-expiratory pressure. Each criterion receives a score from 0 to 4 according to the severity of the condition. The final score was obtained by dividing the collective score by the number of components that were used. A score of zero indicated no lung injury, a score of 1–2.5 indicated mild to moderate lung injury, and a final score of more than 2.5 indicated the presence of ARDS. DPP3 was assessed by measuring once daily 500 μl EDTA whole blood patient samples. DPP3 values below 40 ng/ml were considered as normal; DPP3 values above 40 ng/ml were associated with multi-organ failure and short-term death. Values above 150 ng/ml were depicted as 150 ng/ml and are valid. AT II, angiotensin II; DPP3, dipeptidyl-peptidase 3

**Fig. 2:**
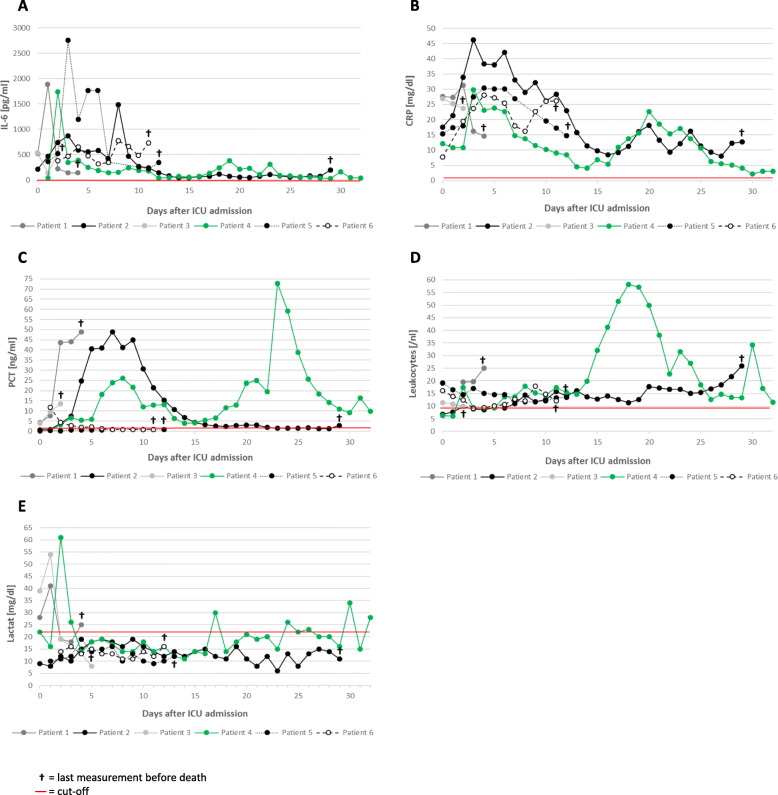
Inflammatory parameters in angiotensin II-treated ICU patients. Assessment of conventional inflammatory parameters in COVID-19-positive ICU patients. For patient-individual angiotensin II treatment interval please refer to Fig. 1. **a** Interleukin 6 (IL-6), **b** C-reactive protein (CRP), **c** procalcitonin (PCT), and **d** leukocytes were measured for each patient upon ICU stay once daily in a routine laboratory. For **e**, lactate concentrations, a blood gas analyzer was used

## Data Availability

Full de-identified dataset and codes of the analyses are available upon request to the corresponding author.
